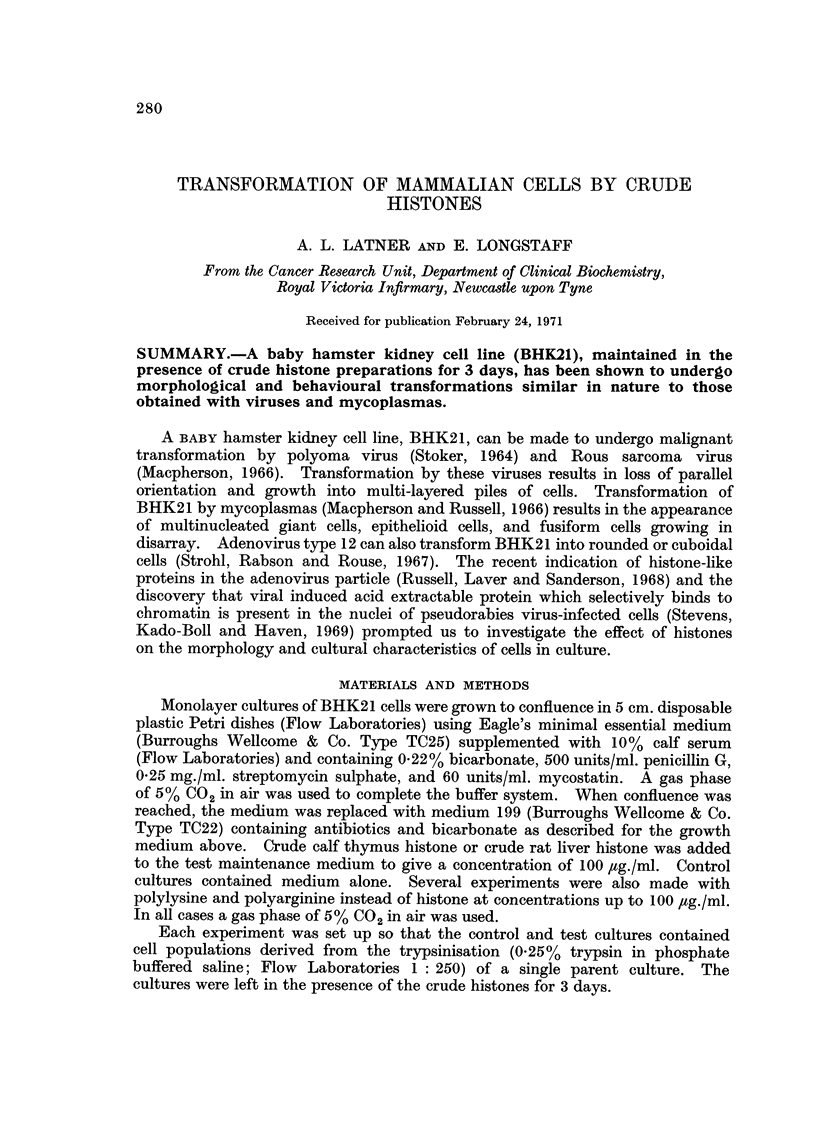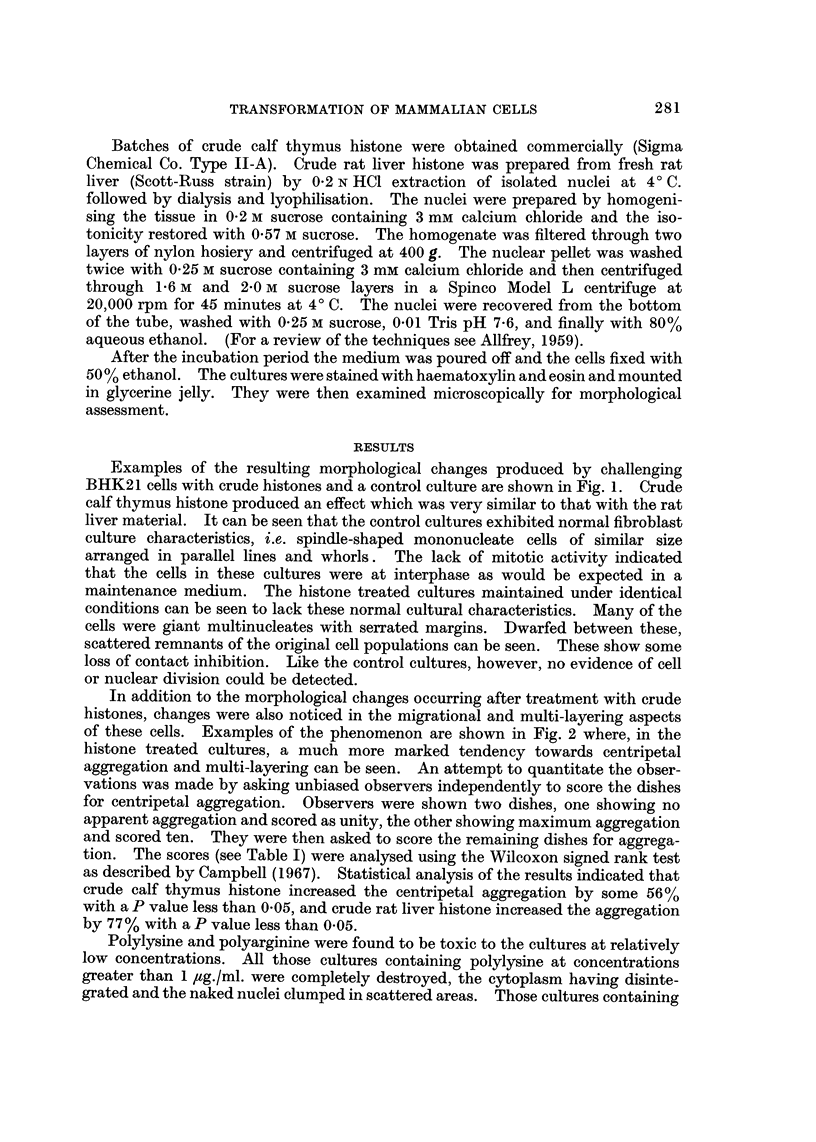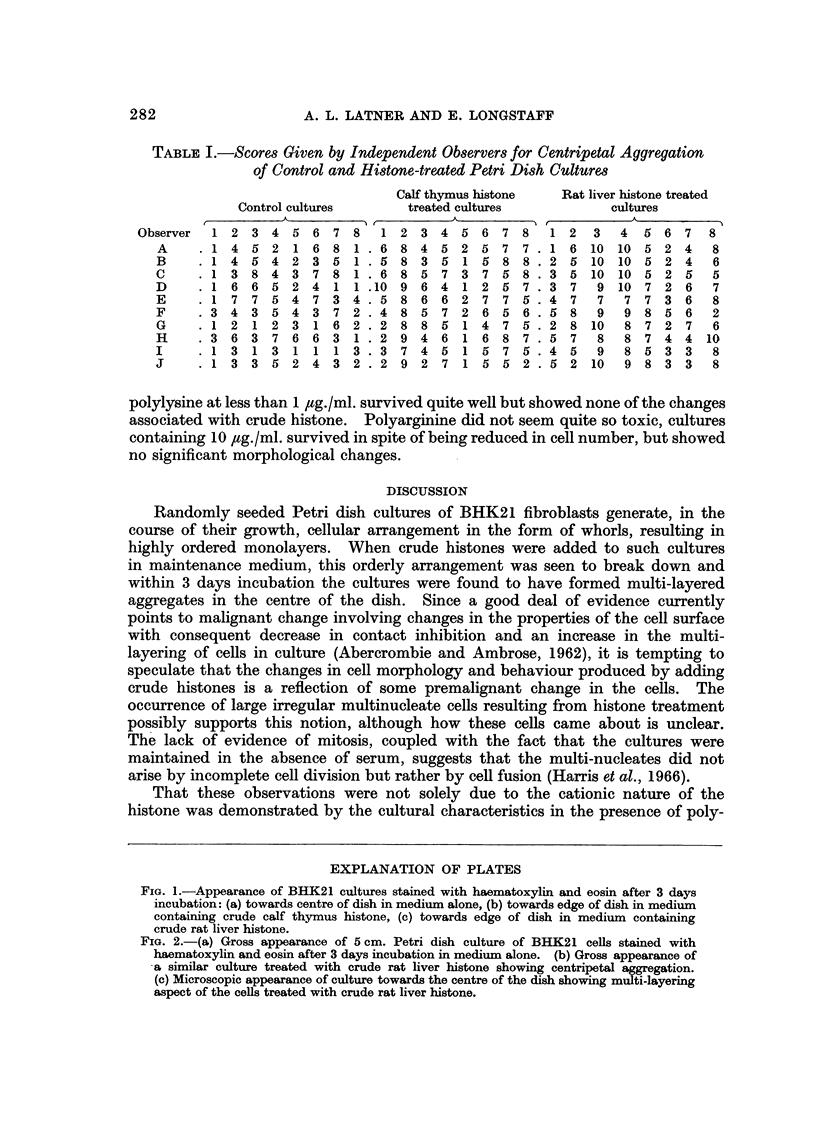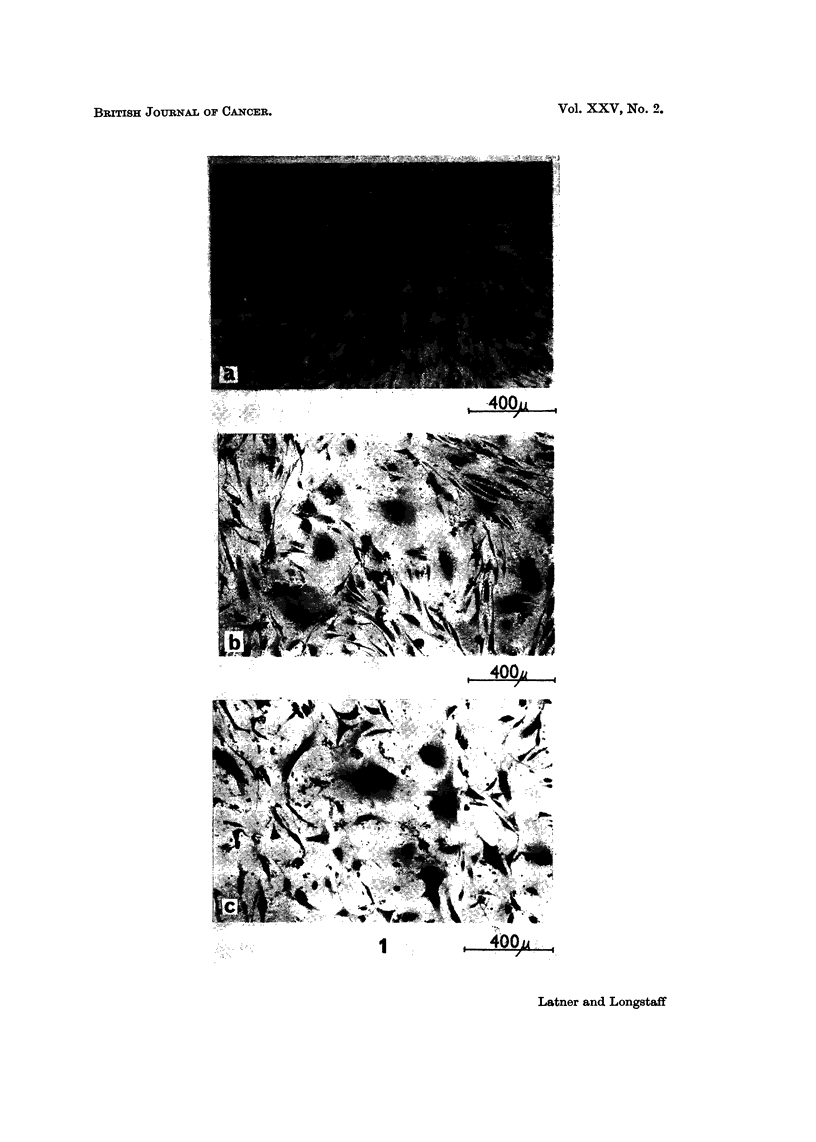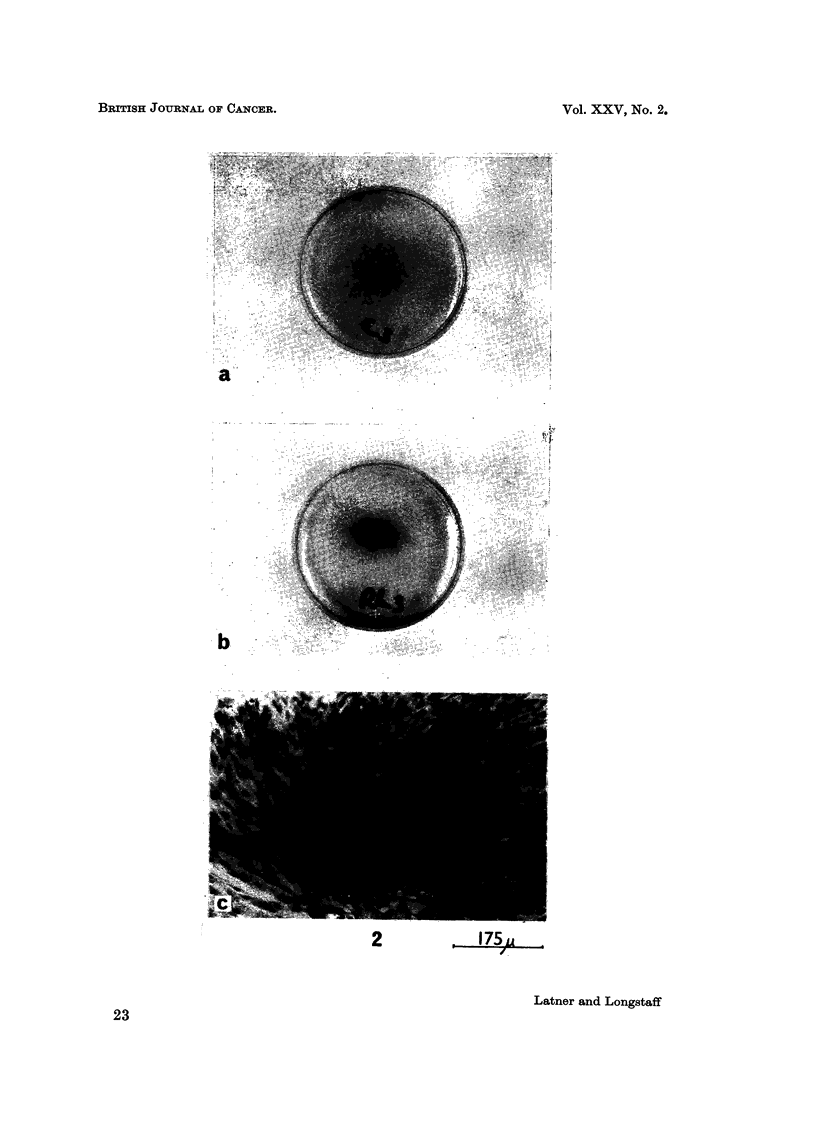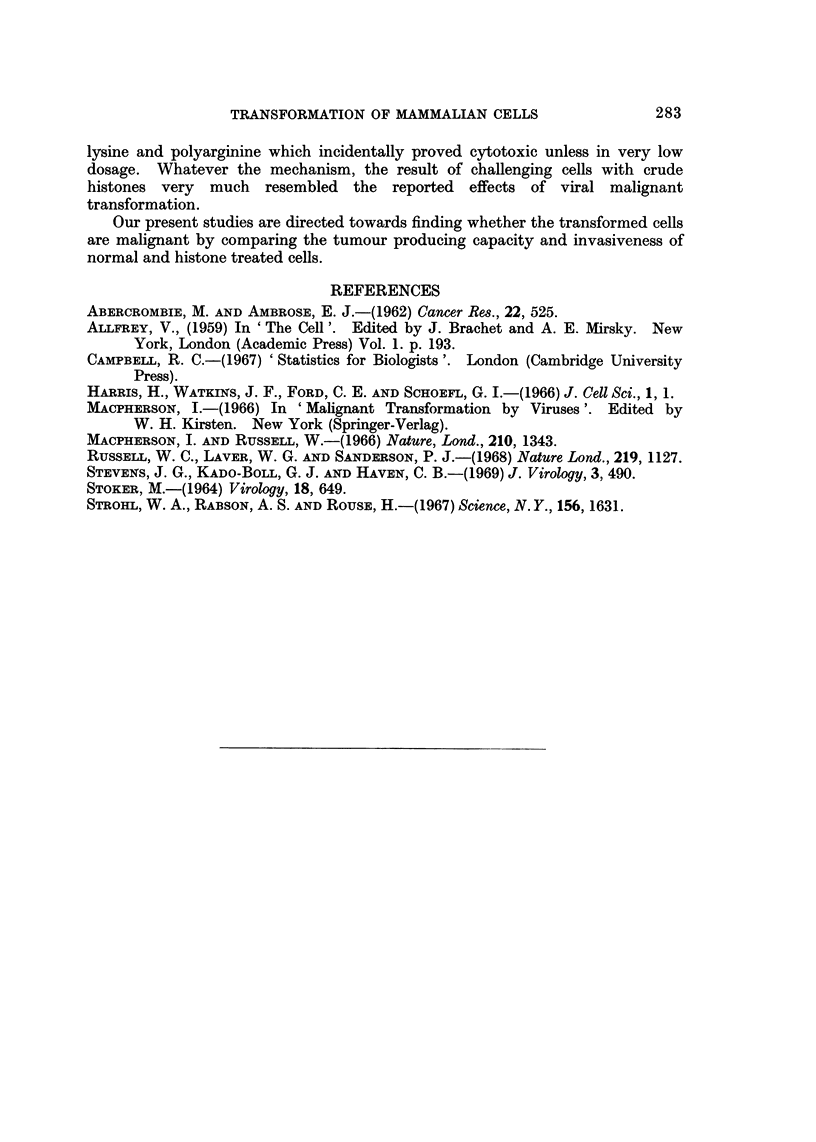# Transformation of Mammalian Cells by Crude Histones

**DOI:** 10.1038/bjc.1971.35

**Published:** 1971-06

**Authors:** A. L. Latner, E. Longstaff

## Abstract

**Images:**


### TRANSFORMATION OF MAMMALIAN CELLS BY CRUDE HISTONES

A. L. LATNER AND E. LONGSTAFF

*From the Cancer Research Unit, Department of Clinical Biochemistry, Royal Victoria Infirmary, Newcastle upon Tyne*

Received for publication February 24, 1971

**SUMMARY.—A baby hamster kidney cell line (BHK21), maintained in the presence of crude histone preparations for 3 days, has been shown to undergo morphological and behavioural transformations similar in nature to those obtained with viruses and mycoplasmas.**

A BABY hamster kidney cell line, BHK21, can be made to undergo malignant transformation by polyoma virus (Stoker, 1964) and Rous sarcoma virus (Macpherson, 1966). Transformation by these viruses results in loss of parallel orientation and growth into multi-layered piles of cells. Transformation of BHK21 by mycoplasmas (Macpherson and Russell, 1966) results in the appearance of multinucleated giant cells, epithelioid cells, and fusiform cells growing in disarray. Adenovirus type 12 can also transform BHK21 into rounded or cuboidal cells (Strohl, Rabson and Rouse, 1967). The recent indication of histone-like proteins in the adenovirus particle (Russell, Laver and Sanderson, 1968) and the discovery that viral induced acid extractable protein which selectively binds to chromatin is present in the nuclei of pseudorabies virus-infected cells (Stevens, Kado-Boll and Haven, 1969) prompted us to investigate the effect of histones on the morphology and cultural characteristics of cells in culture.

### MATERIALS AND METHODS

Monolayer cultures of BHK21 cells were grown to confluence in 5 cm. disposable plastic Petri dishes (Flow Laboratories) using Eagle's minimal essential medium (Burroughs Wellcome & Co. Type TC25) supplemented with 10% calf serum (Flow Laboratories) and containing 0·22% bicarbonate, 500 units/ml. penicillin G, 0·25 mg./ml. streptomycin sulphate, and 60 units/ml. mycostatin. A gas phase of 5% $CO_2$ in air was used to complete the buffer system. When confluence was reached, the medium was replaced with medium 199 (Burroughs Wellcome & Co. Type TC22) containing antibiotics and bicarbonate as described for the growth medium above. Crude calf thymus histone or crude rat liver histone was added to the test maintenance medium to give a concentration of 100 $\mu$g./ml. Control cultures contained medium alone. Several experiments were also made with polylysine and polyarginine instead of histone at concentrations up to 100 $\mu$g./ml. In all cases a gas phase of 5% $CO_2$ in air was used.

Each experiment was set up so that the control and test cultures contained cell populations derived from the trypsinisation (0·25% trypsin in phosphate buffered saline; Flow Laboratories 1 : 250) of a single parent culture. The cultures were left in the presence of the crude histones for 3 days.

Batches of crude calf thymus histone were obtained commercially (Sigma Chemical Co. Type II-A). Crude rat liver histone was prepared from fresh rat liver (Scott-Russ strain) by 0·2 N HCl extraction of isolated nuclei at 4° C. followed by dialysis and lyophilisation. The nuclei were prepared by homogenising the tissue in 0·2 M sucrose containing 3 mM calcium chloride and the isotonicity restored with 0·57 M sucrose. The homogenate was filtered through two layers of nylon hosiery and centrifuged at 400 *g*. The nuclear pellet was washed twice with 0·25 M sucrose containing 3 mM calcium chloride and then centrifuged through 1·6 M and 2·0 M sucrose layers in a Spinco Model L centrifuge at 20,000 rpm for 45 minutes at 4° C. The nuclei were recovered from the bottom of the tube, washed with 0·25 M sucrose, 0·01 Tris pH 7·6, and finally with 80% aqueous ethanol. (For a review of the techniques see Allfrey, 1959).

After the incubation period the medium was poured off and the cells fixed with 50% ethanol. The cultures were stained with haematoxylin and eosin and mounted in glycerine jelly. They were then examined microscopically for morphological assessment.

### RESULTS

Examples of the resulting morphological changes produced by challenging BHK21 cells with crude histones and a control culture are shown in Fig. 1. Crude calf thymus histone produced an effect which was very similar to that with the rat liver material. It can be seen that the control cultures exhibited normal fibroblast culture characteristics, *i.e.* spindle-shaped mononucleate cells of similar size arranged in parallel lines and whorls. The lack of mitotic activity indicated that the cells in these cultures were at interphase as would be expected in a maintenance medium. The histone treated cultures maintained under identical conditions can be seen to lack these normal cultural characteristics. Many of the cells were giant multinucleates with serrated margins. Dwarfed between these, scattered remnants of the original cell populations can be seen. These show some loss of contact inhibition. Like the control cultures, however, no evidence of cell or nuclear division could be detected.

In addition to the morphological changes occurring after treatment with crude histones, changes were also noticed in the migrational and multi-layering aspects of these cells. Examples of the phenomenon are shown in Fig. 2 where, in the histone treated cultures, a much more marked tendency towards centripetal aggregation and multi-layering can be seen. An attempt to quantitate the observations was made by asking unbiased observers independently to score the dishes for centripetal aggregation. Observers were shown two dishes, one showing no apparent aggregation and scored as unity, the other showing maximum aggregation and scored ten. They were then asked to score the remaining dishes for aggregation. The scores (see Table I) were analysed using the Wilcoxon signed rank test as described by Campbell (1967). Statistical analysis of the results indicated that crude calf thymus histone increased the centripetal aggregation by some 56% with a $P$ value less than 0·05, and crude rat liver histone increased the aggregation by 77% with a $P$ value less than 0·05.

Polylysine and polyarginine were found to be toxic to the cultures at relatively low concentrations. All those cultures containing polylysine at concentrations greater than 1 $\mu$g./ml. were completely destroyed, the cytoplasm having disintegrated and the naked nuclei clumped in scattered areas. Those cultures containing

TABLE I.—*Scores Given by Independent Observers for Centripetal Aggregation of Control and Histone-treated Petri Dish Cultures*

| | Control cultures | | | | | | | | Calf thymus histone treated cultures | | | | | | | | Rat liver histone treated cultures | | | | | | | |
|---|---|---|---|---|---|---|---|---|---|---|---|---|---|---|---|---|---|---|---|---|---|---|---|---|
| Observer | 1 | 2 | 3 | 4 | 5 | 6 | 7 | 8 | 1 | 2 | 3 | 4 | 5 | 6 | 7 | 8 | 1 | 2 | 3 | 4 | 5 | 6 | 7 | 8 |
| A | 1 | 4 | 5 | 2 | 1 | 6 | 8 | 1 | 6 | 8 | 4 | 5 | 2 | 5 | 7 | 7 | 1 | 6 | 10 | 10 | 5 | 2 | 4 | 8 |
| B | 1 | 4 | 5 | 4 | 2 | 3 | 5 | 1 | 5 | 8 | 3 | 5 | 1 | 5 | 8 | 8 | 2 | 5 | 10 | 10 | 5 | 2 | 4 | 6 |
| C | 1 | 3 | 8 | 4 | 3 | 7 | 8 | 1 | 6 | 8 | 5 | 7 | 3 | 7 | 5 | 8 | 3 | 5 | 10 | 10 | 5 | 2 | 5 | 5 |
| D | 1 | 6 | 6 | 5 | 2 | 4 | 1 | 1 | 10 | 9 | 6 | 4 | 1 | 2 | 5 | 7 | 3 | 7 | 9 | 10 | 7 | 2 | 6 | 7 |
| E | 1 | 7 | 7 | 5 | 4 | 7 | 3 | 4 | 5 | 8 | 6 | 6 | 2 | 7 | 7 | 5 | 4 | 7 | 7 | 7 | 7 | 3 | 6 | 8 |
| F | 3 | 4 | 3 | 5 | 4 | 3 | 7 | 2 | 4 | 8 | 5 | 7 | 2 | 6 | 5 | 6 | 5 | 8 | 9 | 9 | 8 | 5 | 6 | 2 |
| G | 1 | 2 | 1 | 2 | 3 | 1 | 6 | 2 | 2 | 8 | 8 | 5 | 1 | 4 | 7 | 5 | 2 | 8 | 10 | 8 | 7 | 2 | 7 | 6 |
| H | 3 | 6 | 3 | 7 | 6 | 6 | 3 | 1 | 2 | 9 | 4 | 6 | 1 | 6 | 8 | 7 | 5 | 7 | 8 | 8 | 7 | 4 | 4 | 10 |
| I | 1 | 3 | 1 | 3 | 1 | 1 | 1 | 3 | 3 | 7 | 4 | 5 | 1 | 5 | 7 | 5 | 4 | 5 | 9 | 8 | 5 | 3 | 3 | 8 |
| J | 1 | 3 | 3 | 5 | 2 | 4 | 3 | 2 | 2 | 9 | 2 | 7 | 1 | 5 | 5 | 2 | 5 | 2 | 10 | 9 | 8 | 3 | 3 | 8 |

polylysine at less than 1 μg./ml. survived quite well but showed none of the changes associated with crude histone. Polyarginine did not seem quite so toxic, cultures containing 10 μg./ml. survived in spite of being reduced in cell number, but showed no significant morphological changes.

### DISCUSSION

Randomly seeded Petri dish cultures of BHK21 fibroblasts generate, in the course of their growth, cellular arrangement in the form of whorls, resulting in highly ordered monolayers. When crude histones were added to such cultures in maintenance medium, this orderly arrangement was seen to break down and within 3 days incubation the cultures were found to have formed multi-layered aggregates in the centre of the dish. Since a good deal of evidence currently points to malignant change involving changes in the properties of the cell surface with consequent decrease in contact inhibition and an increase in the multi-layering of cells in culture (Abercrombie and Ambrose, 1962), it is tempting to speculate that the changes in cell morphology and behaviour produced by adding crude histones is a reflection of some premalignant change in the cells. The occurrence of large irregular multinucleate cells resulting from histone treatment possibly supports this notion, although how these cells came about is unclear. The lack of evidence of mitosis, coupled with the fact that the cultures were maintained in the absence of serum, suggests that the multi-nucleates did not arise by incomplete cell division but rather by cell fusion (Harris *et al.*, 1966).

That these observations were not solely due to the cationic nature of the histone was demonstrated by the cultural characteristics in the presence of poly-

### EXPLANATION OF PLATES

FIG. 1.—Appearance of BHK21 cultures stained with haematoxylin and eosin after 3 days incubation: (a) towards centre of dish in medium alone, (b) towards edge of dish in medium containing crude calf thymus histone, (c) towards edge of dish in medium containing crude rat liver histone.

FIG. 2.—(a) Gross appearance of 5 cm. Petri dish culture of BHK21 cells stained with haematoxylin and eosin after 3 days incubation in medium alone. (b) Gross appearance of a similar culture treated with crude rat liver histone showing centripetal aggregation. (c) Microscopic appearance of culture towards the centre of the dish showing multi-layering aspect of the cells treated with crude rat liver histone.

1

Latner and Longstaff

2

lysine and polyarginine which incidentally proved cytotoxic unless in very low dosage. Whatever the mechanism, the result of challenging cells with crude histones very much resembled the reported effects of viral malignant transformation.

Our present studies are directed towards finding whether the transformed cells are malignant by comparing the tumour producing capacity and invasiveness of normal and histone treated cells.

### REFERENCES

Abercrombie, M. and Ambrose, E. J.—(1962) *Cancer Res.*, **22**, 525.

Allfrey, V., (1959) In ' The Cell '. Edited by J. Brachet and A. E. Mirsky. New York, London (Academic Press) Vol. 1. p. 193.

Campbell, R. C.—(1967) ' Statistics for Biologists '. London (Cambridge University Press).

Harris, H., Watkins, J. F., Ford, C. E. and Schoefl, G. I.—(1966) *J. Cell Sci.*, **1**, 1.

Macpherson, I.—(1966) In ' Malignant Transformation by Viruses '. Edited by W. H. Kirsten. New York (Springer-Verlag).

Macpherson, I. and Russell, W.—(1966) *Nature, Lond.*, **210**, 1343.

Russell, W. C., Laver, W. G. and Sanderson, P. J.—(1968) *Nature Lond.*, **219**, 1127.

Stevens, J. G., Kado-Boll, G. J. and Haven, C. B.—(1969) *J. Virology*, **3**, 490.

Stoker, M.—(1964) *Virology*, **18**, 649.

Strohl, W. A., Rabson, A. S. and Rouse, H.—(1967) *Science, N.Y.*, **156**, 1631.